# Factitious Disorder Imposed on Self: Diagnostic Challenges and Clinical Lessons

**DOI:** 10.7759/cureus.102639

**Published:** 2026-01-30

**Authors:** Amy Chen, Tzipora Levitt, Jonathan Shadan, Michael Akhavan, Rachel Siegel, Celeste Defillo-Lopez

**Affiliations:** 1 Medicine, New York Institute of Technology (NYIT) College of Osteopathic Medicine, Old Westbury, USA; 2 Internal Medicine, Icahn School of Medicine at Mount Sinai, Queens Hospital Center, New York, USA

**Keywords:** diagnostic challenges, factitious disorder imposed on self, iatrogenic harm, psychiatry, psychogenic nonepileptic seizures

## Abstract

Factitious disorder imposed on self (FDIS), historically known as Munchausen syndrome, is characterized by the falsification or induction of symptoms in the absence of external incentives. We report the case of a 45-year-old woman with recurrent seizure-like and anaphylaxis-like episodes across multiple hospitalizations. Video electroencephalogram (EEG), laboratory, and imaging studies were inconclusive for both seizures and anaphylaxis. Her clinical course included recurrent unexplained presentations and repeated exposure to potentially invasive interventions despite the lack of objective diagnostic confirmation. This case illustrates the diagnostic challenges of FDIS and highlights the importance of careful clinical evaluation, minimizing iatrogenic harm, and a multidisciplinary management approach.

## Introduction

Factitious disorder imposed on self (FDIS) was first described in 1951 by Richard Asher. It is a psychological disorder in which individuals falsify or induce physical or psychological symptoms [1]. FDIS is rare, affecting approximately 1% of hospitalized patients [2]. This estimate does not reflect confirmed diagnoses and may overestimate prevalence due to diagnostic uncertainty. The pathogenesis of FDIS is unclear; however, psychological factors such as disrupted attachment, abuse, neglect, or trauma are associated with its development. Patients often adopt behaviors consistent with the sick role, typically without identifiable external rewards [3]. 

Diagnostic criteria for FDIS include the following: (1) falsification of physical or psychological signs or symptoms, (2) an individual presents himself or herself to others as ill or injured, and (3) deceptive behavior is evident even in the absence of obvious external rewards. While supportive psychotherapy and cognitive behavioral therapy have shown some benefit in the treatment of FDIS, there are no specific pharmacologic treatments for FDIS [4]. Currently, medical management focuses on avoiding unnecessary, acute interventions, which is relevant to this case.

## Case presentation

A 45-year-old woman with a history of unilateral blindness and hearing loss due to congenital rubella, protein S deficiency, hypothyroidism, obesity, psychogenic nonepileptic seizures (PNES), and multiple prior intubations for threatened anaphylaxis presented to the emergency department (ED) following three consecutive seizure-like episodes at her nursing home, lasting 21, eight, and four minutes, respectively. These episodes were witnessed by nursing home staff, and no tongue biting and incontinence were witnessed during these episodes. She appeared somnolent and confused and returned to her baseline within a few minutes. Initial vital signs were normal, and lab values did not demonstrate elevated prolactin, lactate, or other electrolyte abnormalities, and CT of the head did not demonstrate acute abnormalities.

She was admitted to the intensive care unit (ICU) for monitoring. During the second day of hospitalization, nursing staff reported the patient having asynchronous, abnormal limb movements with transient hypoxia but intermittently withdrew to pain. Due to concern for recurrent seizures, IV midazolam (8 mg) was administered, and her symptoms and mental status returned to baseline after 10 minutes. Arterial blood gas demonstrated respiratory alkalosis (pH 7.54, PCO2 22 mmHg), elevated lactate to 2.4 mmol/L, and elevated prolactin level of 49.4 ng/mL. A portable bedside electroencephalogram (EEG) study concurrent with midazolam was attempted, but it was prematurely terminated after 20 minutes due to the patient removing the electrode band and refusing to proceed with the study. Short-duration EEG has limited sensitivity and does not exclude epilepsy. She later had multiple seizure-like episodes that resolved shortly after the administration of IV saline flushes, and no postictal changes were observed. 

The patient was subsequently transferred to the Medicine service as she remained hemodynamically stable without additional seizure-like episodes. Overnight, she endorsed difficulty breathing following the ingestion of a reported allergen that she had requested, raising concern for inconsistency between reported exposure and clinical findings. Hypotension and urticaria were not witnessed at this time. However, due to observed patient distress and decreasing oxygen saturation, urgent endotracheal intubation was performed for airway protection. Per the intubating physician, there was no laryngeal edema visualized at the time of intubation. The patient returned to the ICU for close monitoring and was extubated the following day as she remained hemodynamically stable and passed a spontaneous breathing trial and leak test. She then began to endorse unilateral upper extremity pain and swelling, which was not appreciated on physical exam, and requested urgent upper extremity imaging to aid in diagnosis. The patient was informed that there would be a wait time for imaging, as there was low suspicion for an acute process. She subsequently chose to leave the hospital against medical advice (AMA). 

Throughout hospitalization, the patient demonstrated the capacity to make medical decisions and refused several interventions, including IV placement and some imaging studies, citing discomfort with invasive procedures. Despite experiencing several seizure-like episodes, some self-reported and others witnessed by medical staff, she refused EEG studies and intermittently refused blood draws to support or help rule out seizure activity. There were also no postictal changes or consistent neurological findings during or following these episodes. 

Since 2019, the patient has had numerous admissions across several hospitals for recurrent episodes of acute respiratory distress and seizure-like activity, frequently reported after food exposure. Her first presentation involved dyspnea and a sensation of throat closure after eating mushrooms, for which she self-administered epinephrine; evaluation, including laryngoscopy, was normal. In subsequent years, she presented with similar complaints, often requiring intubation and treatment for presumed anaphylaxis, despite incongruent clinical findings and the absence of confirmatory allergy testing. Seizure-like episodes were commonly observed during these admissions; however, video EEG studies consistently fail to demonstrate epileptiform activity. Despite extensive negative workups, she did not complete recommended follow-up evaluations, such as 48-hour EEGs, and often left AMA when the treatment plan differed from her expressed preferences, at times returning to the hospital within hours. During one admission, she became agitated during a seizure-like activity that was not treated pharmacologically, prompting placement on 1:1 observation. In this admission, she experienced an episode of tachypnea without hypoxemia or abnormal lung findings, which resolved with albuterol.

This long-standing pattern of recurrent, unexplained presentations, often without clinical evidence and invasive interventions, led to the diagnosis of FDIS. Psychiatry and social work were consulted during this admission, and outpatient mental health follow-up was recommended; however, the patient left the hospital AMA before these services could be coordinated. 

## Discussion

This case describes the presentation of FDIS in one individual and demonstrates the complexity involved in its diagnosis and management. This disorder is historically referred to as Munchausen syndrome and is characterized by the falsification or induction of physical or psychological symptoms in the absence of external reward [5]. Our patient demonstrates some classic characteristics of this condition, including multiple hospital admissions across several years, repeated claims of serious medical conditions such as seizures and anaphylaxis, refusal of diagnostic testing, selective acceptance of treatment, and a pattern of leaving the hospital AMA when the treatment plan differed from clinical recommendations. This patient also underwent multiple video EEGs without any epileptiform activity, and slow limb movements, eye fluttering, and absence of postictal confusion during these studies were observed, which made a diagnosis of epilepsy less likely. On at least one occasion, she was observed holding her breath, resulting in transient hypoxia, which raised concern for a factitious component. 

Although PNES were part of this patient's medical history, PNES and FDIS represent distinct clinical entities despite some overlapping features. PNES is generally understood to be an involuntary manifestation of psychological distress, whereas FDIS involves the falsification or induction of symptoms in the absence of external incentives [6]. In this case, the patient's longitudinal pattern of recurrent unexplained presentations, refusal of diagnostic confirmation, and repeated exposure to high-risk interventions across multiple hospitalizations suggested that her clinical course could not be fully explained by PNES alone, supporting a diagnosis of FDIS.

Diagnosis of FDIS remains largely clinical. According to the Diagnostic and Statistical Manual of Mental Disorders, Fifth Edition, Text Revision (DSM-5-TR), three criteria must be met: (1) falsification of symptoms, (2) presentation of oneself as ill or injured, and (3) absence of obvious external rewards [1]. In this case, all three criteria are fulfilled. The patient demonstrated a recurrent pattern of behaviors consistent with assuming the sick role, including repeated hospitalizations and undergoing invasive interventions, such as intubation and administration of benzodiazepines. She frequently declined less invasive investigations, such as EEG or imaging, while simultaneously accepting or requesting more invasive procedures. 

This case also highlights the potential risk of iatrogenic harm in patients presenting with recurrent symptoms that would require invasive interventions. In this patient, repeated endotracheal intubations were performed for impending respiratory failure and airway protection, yet no laryngeal edema was found. Such interventions expose patients to avoidable risks, including airway trauma, sedation-related complications, and prolonged hospitalization. These findings underscore the dangerous situations that patients knowingly expose themselves to, as well as the importance of careful clinical assessment and diagnostic verification to minimize harm while ensuring patient safety.

Management of patients with FDIS requires a sensitive approach. Direct confrontation is usually discouraged, as it may lead to the patient disengaging from care or exacerbating maladaptive behaviors. Instead, a consistent, non-reinforcing, and team-based approach is recommended [7]. Unnecessary testing or treatments should be avoided to reduce the reinforcement of maladaptive behaviors. In this case, the neurology team appropriately advised against the use of benzodiazepines for recurrent seizure-like episodes, as they were most likely pseudoseizures, given the absence of EEG, laboratory, and clinical support for diagnosis [8,9]. Benzodiazepines also increase the risk of respiratory depression and iatrogenic harm in nonepileptic events. 

Interdisciplinary collaboration is essential in managing cases of FDIS, with psychiatry, neurology, social work, and primary care developing a management plan that limits unnecessary interventions while providing support and structure [5]. Although patients with FDIS may initially refuse psychiatric care, early involvement of mental health professionals can help decrease recurrent hospital visits and prevent unnecessary harm from medical treatments [10]. In addition, specifying airway management and sedative administration are pertinent, as these are primary medical risks illustrated in this case report. For patients who move from hospital to hospital to avoid being identified as "frequent visitors," it is especially important to maintain consistent care and close follow-up [11]. 

Ultimately, the prognosis for patients with FDIS remains uncertain, as many patients continue to have recurrent hospitalizations despite treatment efforts. However, some improvement can be made through consistent, organized care, setting clear boundaries, and involving psychiatric services early on [12]. 

## Conclusions

The case highlights FDIS presenting with recurrent seizure-like episodes and frequently reported anaphylactic episodes and underscores the importance of maintaining a high index of suspicion for FDIS in patients with recurrent, unexplained, or inconsistent clinical presentations. A comprehensive, multidisciplinary approach encompassing psychiatry, neurology, and primary care is essential for accurate diagnosis, the minimization of unnecessary interventions, and ensuring appropriate management. Additionally, this case emphasizes the need for careful assessment and verification of acute conditions such as seizures and life-threatening anaphylaxis prior to initiating invasive and/or long-term medical therapies to prevent potential harms and optimize outcomes.
